# How are socioeconomic status, social support, and health history associated with unhealthy lifestyle behaviours in middle-aged adults? Results of the Swedish CArdioPulmonary bioImage Study (SCAPIS) COHORT

**DOI:** 10.1186/s13690-025-01513-7

**Published:** 2025-03-24

**Authors:** Leonie Klompstra, Marie Löf, Cecilia Björkelund, Mai-Lis Hellenius, Lena V. Kallings, Marju Orho-Melander, Patrik Wennberg, Preben Bendtsen, Marcus Bendtsen

**Affiliations:** 1 Department of Health, Medicine and Caring Sciences, Linköping University, Linköping, Sweden; 2 Department of Biosciences and Nutrition, Karolinska Institutet, Stockholm, Sweden; 3 Primary Health Care/Department of Public Health and Community Medicine, Institute of Medicine, Sahlgrenska Academy, University of Gothenburg, Gothenburg, Sweden; 4 Research, Education, Development & Innovation, Primary Health Care, Region Västra Götaland, Gothenburg, Sweden; 5 Department of Medicine, Karolinska Institutet, Stockholm, Sweden; 6 Department of Physical Activity and Health, The Swedish School of Sport and Health Sciences, Stockholm, Sweden; 7 Family Medicine and Preventive Medicine, Department of Public Health and Caring Sciences, Uppsala University, Uppsala, Sweden; 8 Department of Clinical Sciences, Lund University, Malmö, Sweden; 9 Department of Public Health and Clinical Medicine, Family Medicine, Umeå University, Umeå, Sweden; 10 Department of Medical Specialist in Motala, Linköping University, Linköping, Sweden

**Keywords:** Unhealthy lifestyle behaviour, Middle-aged adults, Alcohol consumption, Physical inactivity, Diet, Smoking

## Abstract

**Background:**

Unhealthy lifestyle behaviours, including unhealthy alcohol consumption, physical inactivity, smoking, and nonadherence to dietary recommendations, are major contributors to non-communicable diseases and mortality. While adopting healthy behaviours can reduce these risks in middle-aged adults, research is limited. Therefore, the aim of this study was to assess the distribution of unhealthy lifestyle behaviours in middle-aged adults and their associations with socioeconomic factors, social support, and history of disease.

**Method:**

This was a cross-sectional study of the Swedish CArdioPulmonary bioImage Study (SCAPIS) cohort (2013–2018) at six Swedish university hospitals. Multilevel regression models were estimated using Bayesian inference with priors shrinking covariate estimates towards the null.

**Results:**

In total, 59 909 participants, aged 50–64 years old were invited to SCAPIS, of which 30 154 (50.3%) decided to participate. The mean age of participants was 58 (SD = 4) years old, and 51% were women (n = 15 508). Men had higher unhealthy alcohol consumption and were less adherent to dietary recommendations compared to women. Older participants were more physically inactive compared to younger participants.

Low education and financial difficulties were associated with smoking, physical inactivity, and poor diet adherence. Financial difficulties were also associated with unhealthy alcohol consumption. Having more people to turn to in difficulties was associated with lower alcohol consumption. Lack of appreciation and comfort support was associated with smoking and poor diet adherence. Diabetes was associated with lower alcohol consumption. Diabetes and lung diseases were associated with smoking and inactivity.

**Conclusions:**

Middle-aged adults with lower socioeconomic status, less quality social support, and a history of disease were more likely to engage in unhealthy behaviours. This study helps to identify groups of middle-aged adults who may require additional attention when it comes to prioritizing the development of preventive measures.

**Supplementary Information:**

The online version contains supplementary material available at 10.1186/s13690-025-01513-7.

**Table:** 

**Textbox 1. Contributions to the literature**	
• There is limited research on unhealthy lifestyle behaviours in middle-aged adults.	
• By examining socioeconomic factors, social support, and medical history together, the study provides a holistic view of which factors are associated with unhealthy behaviours in middle-aged adults.	
• Unhealthy lifestyle behaviours, such as smoking and poor diet adherence, are unevenly distributed across different social and economic backgrounds, highlighting the need for targeted health interventions.	
• Social support can both positively and negatively influence health behaviours, deepening our understanding of its impact.	

## Introduction

Unhealthy lifestyle behaviours, including unhealthy alcohol consumption, physical inactivity, smoking, and non-adherence to dietary recommendations, are among the leading causes of non-communicable diseases—including cardiovascular disease, cancer and diabetes [1–3]. Over the past decade, adherence to a healthy lifestyle has decreased [4, 5], except for a notable reduction in smoking prevalence [6]. Research on unhealthy lifestyle behaviours has largely been concentrated in the early half of the life course, namely, childhood, adolescence and early and young adulthood [7, 8], and in older adults [9, 10]. However, the literature concerning unhealthy lifestyle behaviours in middle-aged adults is limited [11].

Middle age holds considerable significance in life's journey, as it marks not only the commencement of the concluding phase of one's professional accomplishments but also stands as the threshold before the onset of old age and physical decline that follows [11].

Research in middle-aged adults shows that adherence to healthy behaviours could reduce the risk of non-communicable diseases and all-cause mortality [12, 13], and middle-aged adults who newly adopt a healthy lifestyle experience benefits of lower rates of cardiovascular disease and mortality [14]. This emphasizes that to minimize the risk of poor health in later life, there is a need for effective strategies to change middle-aged adults’ behaviour. It is, therefore, important to identify factors associated with unhealthy lifestyle behaviours, as this allows for targeted interventions and the development of evidence-based strategies. This study aimed to assess the distribution of unhealthy lifestyle behaviours in middle-aged adults and to estimate conditional associations between unhealthy lifestyle behaviours with biological sex, age, socioeconomic factors, social support, and history of disease.

## Methods

This study was a cross-sectional study based on the baseline data from the Swedish CArdioPulmonary bioImage Study (SCAPIS) [15], which is a Swedish nationwide population-based cohort study mainly designed to research cardiovascular and chronic obstructive pulmonary diseases. The SCAPIS data collection was carried out between 2013 and 2018 as a multicentre study at Swedish university hospitals. The participants visited the university hospital on two to three occasions within a two-week period. During the first visit, the participant filled in an extensive questionnaire on lifestyle behaviours. In total, 59 909 individuals were invited to SCAPIS, of which 30 154 (50.3%) decided to participate.

### Outcomes and measures

Participants of SCAPIS were asked to complete a questionnaire including questions on biological sex, age, socioeconomic factors, social support, history of disease, family history of disease, alcohol consumption, smoking, physical inactivity, and intake of food and drinks.

*Socioeconomic factors* assessed were educational level, employment status, marital status, ability to find 1800 Euros in a week for unforeseen events, difficulties managing regular expenses, living situation, region of birth, and parents’ region of birth.

*Social support* was assessed with a validated condensed version of the Interview Schedule for Social Interaction [16]. In this study, the twelve items in the condensed version were analysed separately.

*Self-reported history of diseases* assessed were: myocardial infarction, angina pectoris, atrial fibrillation, heart failure, or heart valve disease, coronary artery bypass graft surgery (CABG), percutaneous coronary intervention (PCI) intervention, peripheral artery disease intervention, aortic intervention, stroke, hypertension, hyperlipidemia, diabetes, chronic obstructive pulmonary disease (COPD), chronic bronchitis, emphysema, tuberculosis, or other lung diseases, asthma, sleep apnea, celiac disease, Crohn’s disease, ulcerative colitis, rheumatic disease, and cancer. Self-reported history of diseases was not validated to objective measures.

*Family history of disease* was assessed by asking about any first-degree relative’s history of diabetes, asthma, and bronchitis. Further, participants were asked about parents’ or siblings’ history of disease of myocardial infarction, stroke, and lung cancer.

*Unhealthy alcohol consumption* was measured using the alcohol use disorders identification test (AUDIT) [17]. The answer options in the last two questions typically have three response alternatives (No; Yes, but not in the last year; Yes; during the last year), but in the SCAPIS study, only two response alternatives were used (No; Yes). The total score of AUDIT ranges between 0 and 40 points, with higher scores indicating more harmful alcohol use. Typically, 8 points or more indicates hazardous or harmful alcohol consumption.

*Smoking* was assessed with a single question: “Do you smoke?”. The answer alternatives were: No, I never smoked; No, I stopped smoking; Yes, smoking occasionally; Yes, smoking regularly; Not willing/able to reply. Patients who stated that they were current smokers when asked orally during a study visit where classified as smokers regardless of their response to the questionnaire.

*Physical inactivity* was defined by the World Health Organization as doing insufficient physical activity to meet current physical activity recommendations [18]. The recommendation for adults aged 18–64 years old is doing at least 150–300 min of moderate-intensity aerobic physical activity or at least 75–150 min of vigorous-intensity aerobic physical activity; or an equivalent combination of moderate- and vigorous-intensity activity throughout the week. They should also do muscle-strengthening activities at moderate or greater intensity that involve all major muscle groups on two or more days a week. Physical inactivity measured with a single question assessing lack of exercise: “How often have you been training or exercised in training clothes over the last three months, in order to improve your endurance and/or to feel good?” The answer alternatives were: Never; Sometimes, but not regularly; 1–2 times week; 2–3 times a week; More than 3 times a week; Not willing/able to reply. The answer alternatives “Never” or “Sometimes, but not regularly” indicated physical inactivity.

*Non-adherence to dietary recommendations* was assessed by food intake, measured with the MiniMeal-Q [19]. This questionnaire includes 75 to 126 food items and asks about dietary intake during the past few months. The intakes of energy (kJ/day) and macronutrients (g/day) were calculated using the national database on nutrient content published by the Swedish National Food Agency. The Swedish Healthy Eating Index score (SHEI-score) was used to determine non-adherence to dietary recommendations. The index is a ratio between the recommended consumption of nine foods and nutrient intake included in the 2012 Nordic Recommendations.

The score for each of the nine foods and nutrient intake ranges from 0 to 1, and the total score ranges from 0 to 9, with a lower score indicating lower adherence to the dietary recommendations. Note that, for coherence with the other measures of behaviours, we reversed the scale in the enclosed analyses so that higher scores indicated lower adherence and vice versa.

### Data analyses

A protocol, including a statistical analysis plan, was registered on the Open Science Framework before we started the analysis [20]. We used multilevel regression with random intercepts for site to estimate both the distribution of behaviours in the entire cohort, and associations between behaviour and socioeconomic factors, social support, and history of disease. We used negative binomial regression for AUDIT scores since this measure is truncated at zero and typically heavily left-skewed. Linear regression was used for SHEI-scores since the measure is typically not skewed and conditional errors can be considered normally distributed, finally, we used logistic regression for the two binary outcomes of physical inactivity and smoking.

We used Bayesian inference to estimate the regression models. In this paradigm, the focus is on estimating a distribution of estimates of associations, known as the *posterior* distribution. The posterior distribution provides evidence for the direct question of how likely it is that an association is greater or less than the null. In contrast, null hypothesis testing assumes that the association is the null and then tests the indirect question regarding the extremeness of the data under this assumption. In the Bayesian paradigm, no null hypothesis test is made since it does not respond to the dichotomous questions about the existence or non-existence of an association, but rather, it speaks towards how likely different values of an association are conditional on our prior beliefs and the data collected. So called *prior distributions* are used to encode beliefs before the data, which can be much more conservative than in typical null hypothesis testing.

We reported posterior distributions of regression model parameters, with medians representing point estimates of incidence rate ratios (IRR), odds ratios (OR), and mean differences (MD), along with 50% and 95% compatibility intervals (CI) defined by the percentiles of the posterior distributions.

To estimate the distribution of unhealthy lifestyle behaviours in the entire cohort we omitted covariates from the models. To estimate conditional associations between behaviours and socioeconomic factors, social support, and history of disease, we estimated the distribution of each behaviour measure with the three adjustment sets respectively. These analyses estimate the conditional associations between the adjustment variables and the behaviour, displaying their relative contribution in explaining behaviour. We used standard normal priors for random and fixed intercepts with a wider normal prior in linear regression models (mean = 0, SD = 10). To account for the excessive number of covariates in the regression models, we encoded a strong prior conservative belief by using Cauchy priors with a half-normal hyperprior for the scale parameter. This induced sparsity in the model since estimates were strongly pulled towards the null and only coefficients representing strong associations in the data escaped this so-called *shrinkage* of the model. Data were analysed using the R statistical software version 4·0·4 and Stan 2.30.1 (CmdStan).

## Results

In Table 1, the characteristics of the participants are presented. The mean age of participants was 58 (SD = 4) years old, and 51% were women (n = 15 508). The median posterior AUDIT score in the cohort was 3.76 (95% CI = 3.4; 4.1), and the median posterior Swedish Healthy Eating Index score (reversed) was 5.99 (95% CI = 5.7; 6.2). The median posterior prevalence of current smokers was 12.8% (95% CI = 9.2%; 18.8%) and the median posterior prevalence of physical inactivity was 50% (95% CI = 46.3%; 53.5%).

**Table 1:** Demographics, socio-economic factors, social interaction, social support, self-reported history of disease and family history of disease of 30,154 participants aged 50 to 65

**Demographics**	
**Age (mean, sd)**	**58 (4)**
**Women**	**51% (15,508)**
**Socio-economic factors**	
**Education**	
No education	0.7% (197)
Primary school	9% (2550)
Secondary school	44% (13,335)
University	44% (13,218)
Missing	3% (854)
**Employment status**	
Gainfully Employed	81% (24,483)
Early retirement or sickness pension	6% (1654)
Old age or contractual pensioner	5% (1535)
Unemployed or labour market measures	4% (1072)
Other not gainfully employed (including leave of absence, parental leave, studying or training)	3% (829)
Long term sick listed (more than 3 months)	2% (681)
**Ability to find 1 800 EUR in a week**	88% (26,540)
**Difficulties managing expenses**	5% (1608)
**Type of residence**	
Own house	45% (13,677)
Own apartment	29% (8712)
Rental Apartment	22% (6691)
Other	0.8% (244)
Missing	3% (830)
**Marital status**	
Married	71% (21,514)
Alone	13% (3975)
Divorced	11% (3275)
Widow	2% (491)
Missing	0.6% (899)
**Living situation**	
With spouse or partner	71% (21,403)
With children	28% (8281)
Alone	19% (5651)
With parents, sibling, or other adults	2% (719)
**Region of birth**	
European	91% (27,519)
Eastern Mediterranean	3% (1017)
Americans	1% (401)
African/Southeast Asian/Western Pacific	1% (373)
Missing	3% (844)
**Region of birth father**	
European	87% (26,077)
Eastern Mediterranean	3% (948)
Americans	1% (411)
African/Southeast Asian/Western Pacific	1% (372)
Missing	8% (2346)
**Region of birth Mother**	
European	87% (26,155)
Eastern Mediterranean	3% (941)
Americans	1% (394)
African/Southeast Asian/Western Pacific	1% (358)
Missing	8% (2306)
**Social Interaction**	
Number acquaintances with shared interests (mean, sd)	3 (1)
Number people met during an ordinary week (mean, sd)	3 (1)
Number people who can easily be asked for assistance (mean, sd)	3 (1)
Number of friends that you can visit any time (mean, sd)	2 (1)
Number of people with whom speak openly (mean, sd)	2 (1)
Number of people who to turn to in difficulties (mean, sd)	2 (1)
**Social support**	
Tangible support	89% (26,843)
Person to share feelings of happiness	88% (26,561)
Person who is close	86% (25,869)
Person to confide	84% (25,365)
Person who appreciates your efforts	84% (25,459)
Person to comfort	75% (22,523)
**Self-reported history of disease**	
Hypertension	22% (6620)
Hyperlipidemia	11% (3425)
Asthma	8% (2422)
Cancer	6% (1738)
Myocardial infarction, angina pectoris, atrial fibrillation, heart failure, or heart valve	5% (1436)
Sleep apnea	4% (1292)
Diabetes	4% (1291)
Rheumatic disease	4% (1083)
Chronic obstructive pulmonary disease, chronic bronchitis, emphysema, tuberculosis, or other lung diseases	3% (804)
CABG, PCI intervention, peripheral artery disease intervention, or aortic intervention	1% (428)
Stroke	1% (421)
Crohn's disease or ulcerative colitis	1% (325)
Celiac disease	0.6% (186)
**Self-reported family history of disease**	
Myocardial infarction	27% (8199)
Diabetes	26% (7929)
Stroke	26% (7766)
Asthma	21% (6365)
Bronchitis, Chronic obstructive pulmonary disease or emphysema	15% (4465)
Lung cancer	7% (2032)

**Fig. 1:**
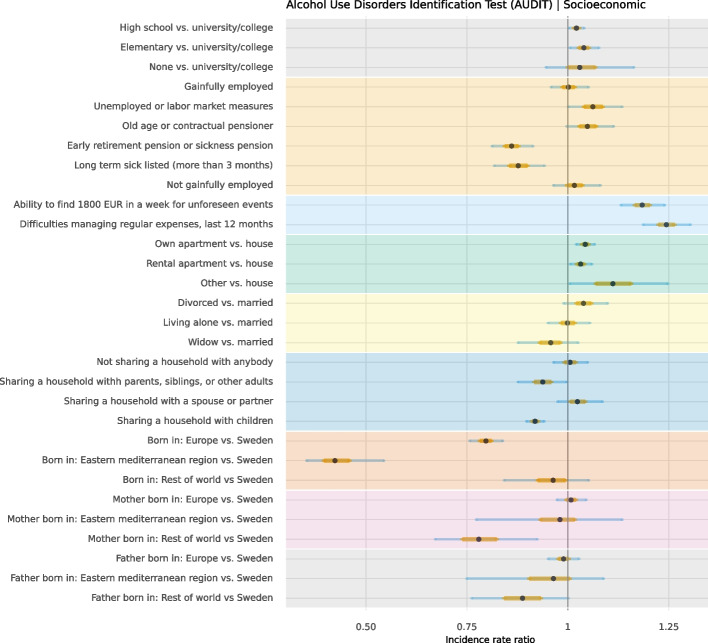
Distribution of unhealthy alcohol consumption, smoking, physical inactivity, and adherence to dietary recommendations in socioeconomic factors in 30,154 participants aged 50 to 65

**Fig. 2:**
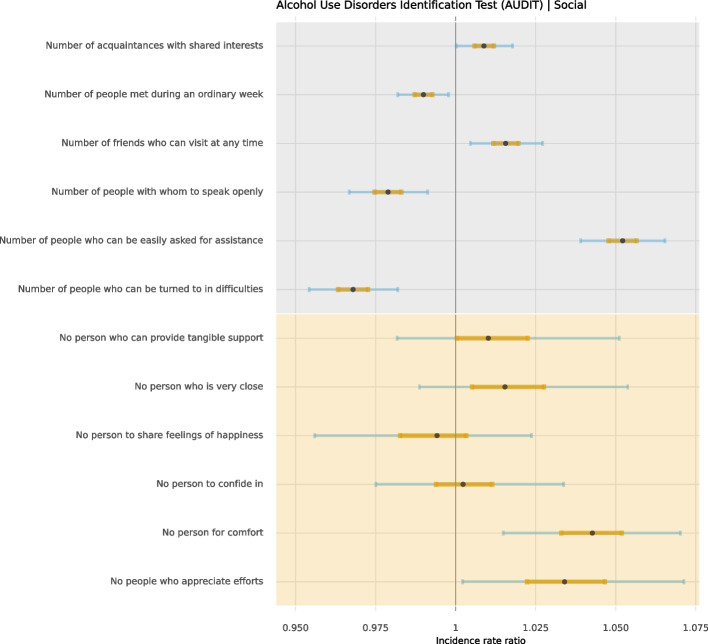
Distribution of unhealthy alcohol consumption, smoking, physical inactivity, and adherence to dietary recommendations in social support in 30,154 participants aged 50 to 65

**Fig. 3:**
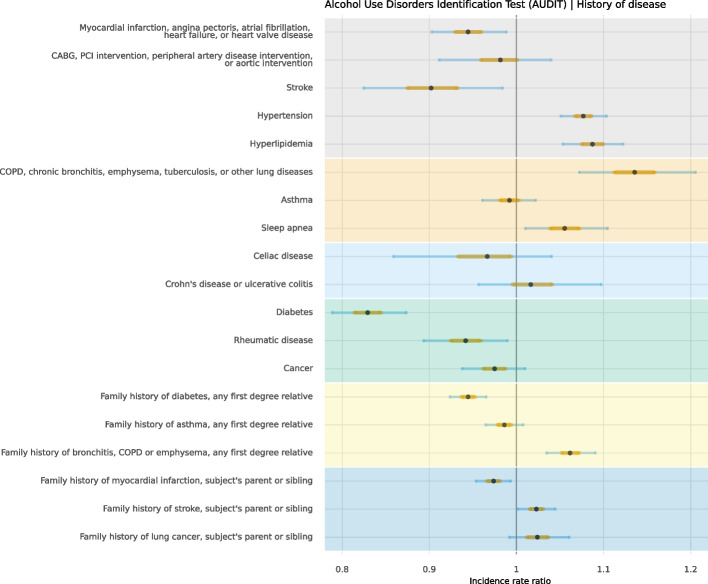
Distribution of unhealthy alcohol consumption, smoking, physical inactivity, and adherence to dietary recommendations in self-reported history of support and family history of disease in 30,154 participants aged 50 to 65

Point estimates and CIs of conditional associations are presented for: (1) behaviour and socioeconomic factors in Fig. 1 and Suppl. 1; (2) behaviour and social support in Fig. 2 and Suppl. 2; and (3) behaviour and history of disease in Fig. 3 and Suppl. 3. Across analyses of the three domains, findings suggested that men reported a higher degree of unhealthy alcohol consumption and were more often non-adherent to dietary recommendations compared to women. Also, older participants were more likely to be physically inactive compared to younger participants.

### Socioeconomic factors

Participants who only finished elementary school or high school were more likely to be current smokers (elementary school OR = 2.58, CI 95% = 2.26; 2.93; high school OR = 1.71, CI 95%, 1.57; 1.86) and be physically inactive (elementary school OR = 2.23, CI 95% = 2.02; 2.47; high school OR = 1.57; CI 95%, 1.49; 1.66) compared to participants who had a university or college degree. These participants were also less adherent to dietary recommendations (elementary school MD = 0.50, CI 95% = 0.45; 0.56; high school MD = 0.29, CI 95% = 0.26; 0.32).

Participants who had difficulties managing their expenses in the last year had a higher degree of unhealthy alcohol consumption (IRR = 1.24, CI 95% = 1.19; 1.30), were more likely to be current smokers (OR = 1.41; CI 95% = 1.20; 1.64) and physically inactive (OR = 1.56, CI 95% = 1.37; 1.79) and were less adherent to dietary recommendations (MD = 0.10, CI 95% = 0.03; 0.17). The ability to find 1800 Euro in a week for unforeseen events was associated with a higher degree of unhealthy alcohol consumption (IRR = 1.18, CI 95% = 1.13; 1.24), but on the other hand, was associated with lower odds of smoking (OR = 0.68, CI 95% = 0.59; 0.79) and greater adherence to dietary recommendation (MD = -0.18, CI 95% = -0.25; -0.11).

### Social support

Participants with more people who could easily be asked for assistance had a higher degree of unhealthy alcohol consumption (IRR = 1.05, CI 95% = 1.04; 1.07). However, participants with more people they could turn to in difficulties reported lower unhealthy alcohol consumption (IRR = 0.97, CI 95% = 0.95; 0.98). When participants felt they had no person to turn to for comfort, unhealthy alcohol consumption was again conditionally higher (IRR = 1.04; CI 95% = 1.01; 1.07). These participants were also less adherent to dietary recommendations (MD = 0.14; CI 95% = 0.09; 0.19).

Those with more acquaintances with shared interests were less likely to be physically inactive (OR = 0.90, CI 95% = 0.97; 0.93). Participants with the non-existence of people who appreciate their efforts were more likely to be current smokers (OR = 1.30, CI 95% = 1.14; 1.47) and physically inactive (OR = 1.17, CI 95% = 1.06; 1.29). These participants were also less adherent to dietary recommendations (MD = 0.14).

### Self-reported history of disease and family history of disease

Participants with a history of diabetes reported a lower degree of unhealthy alcohol consumption (IRR = 0.83, CI 95% = 0.79; 0.87) but were more likely to be physically inactive (OR = 1.43; CI 95% = 1.24; 1.64) and current smokers (OR = 1.29, CI 95% = 1.04; 1.57). Participants with a history of hypertension and sleep apnoea were more likely to be physically inactive (hypertension OR = 1.31, CI 95% = 1.23, 1.40; sleep apnoea OR 1.36, CI 95% = 1.19, 1.55). Finally, participants with a history of a lung disease were more likely to be smokers (OR = 1.99, CI 95% = 1.63, 2.40), to be physically inactive (OR = 1.49, CI 95% = 1.26, 1.78) and were less adherent to dietary recommendation (MD = 0.19, CI 95% = 0.09, 0.29).

## Discussion

In a cohort of randomly sampled individuals aged 50–65 years old, we found that the prevalence of unhealthy lifestyle behaviours was unequally distributed concerning biological sex, age, socioeconomic factors, social support, and history of disease.

### Biological sex and age

Men had higher unhealthy alcohol consumption compared to women, which is a well-known pattern of behaviour [21]. Likewise, men were more often non-adherent to dietary recommendations compared to women, which could be explained by differences in body image perceptions and the use of dieting to lose weight [22]. Our study also found that, among middle-aged adults, older age was associated with greater physical inactivity, a finding also confirmed in a sub-analysis of the SHARE data, which showed that physical inactivity increased with age [23].

### Socioeconomic factors

Middle-aged adults with lower education and financial struggles were more prone to smoking, inactivity, and poor diet adherence. Financial struggles were also associated with higher unhealthy alcohol behaviour. Associations between healthy behaviours and educational level have been shown previously, with higher educational level positively relating to not smoking [24], physical activity levels [25], and adherence to dietary recommendations [26, 27]. Previous studies have also shown that high educational levels and income in adulthood may represent protective factors against harmful alcohol use [28, 29]. Our study showed that being able to access €1800 for unforeseen events increased unhealthy alcohol consumption but decreased smoking and improved dietary adherence. An association between higher socioeconomic status and increased episodes of heavy drinking has been found in Finland (a neighbouring country to Sweden) [30], suggesting that socioeconomic factors may not protect against harmful alcohol use but may rather induce different drinking patterns.

### Social support

It has previously been found that middle-aged and older adults who feel alone were less frequent drinkers [31], and that social networking with male friends was associated with higher alcohol consumption [32]. This is congruent with our findings that those with more people who can easily be asked for assistance reported increased levels of unhealthy alcohol consumption. On the other hand, it has also been observed that social support at the community level has a protective effect against heavy episodic drinking [32], which is in line with our findings that those who felt that they had no person to turn to for comfort, and fewer people to turn to in difficulties, reported increased levels of unhealthy alcohol consumption.

Studies have found that individuals who perceive insufficient quality or quantity of social connections were likelier to smoke and have poorer dietary habits [33–35]. Our study found that feeling unappreciated or lacking someone for comfort was associated with higher smoking rates and lower adherence to dietary recommendations. Having acquaintances with shared interests and feeling appreciated reduced the likelihood of physical inactivity. This is in line with previous studies that have shown that older adults who felt social support for being physically active were more likely to do leisure-time physical activity [36].

### History of disease

Participants diagnosed with diabetes reported less unhealthy alcohol behaviour, but on the other hand, were more likely to be current smokers and physically inactive. Physical inactivity was also reported at a higher proportion among those with hypertension. These patterns of behaviour among patients with diabetes and hypertension have also been observed before [37, 38]. Participants suffering from a lung disease were more likely to be current smokers, physically inactive, and non-adherent to dietary recommendation. Although COPD is by far the most common smoking-associated lung disease, other rare pulmonary diseases have been linked to smoking [39]. In patients with COPD, it has been found that physical activity substantially decreases across all severity stages of the disease [40].

### Strength and limitations

The strengths of this study include the large sample size and the cohort being representative of the Swedish general population, which strengthens the generalisability [41]. This is also the first study which examined the unhealthy lifestyle behaviours among middle-aged adults.

This study also has several limitations that should be considered when interpreting findings. First, the design is cross-sectional, and the analysis methods produce conditional associations which should not be interpreted as estimates of causal effects. Further, associations can be induced by controlling for other factors, i.e., through collider bias, and so estimates of associations should be interpreted as conditional on all other covariates in the models. This does not reduce their usefulness in identifying individuals prone to unhealthy behaviours and may benefit from interventions. However, the study cannot represent the societal benefits of intervening in these populations, nor the magnitude of effect required for long-term health outcomes to be beneficial. This requires detailed health economic evaluations, which we leave for future work.

Although not the primary purpose of this study, it is limited in its ability to explain why the associations found exists. Socio-demographic inequalities continue to burden society due to behavioural determinants of health not being equally distributed; however, why this continues to be the case is beyond the scope of this study. Finally, the variables analysed are all self-reported, which makes them prone to being biased, especially considering the study context in which participants responded to the questionnaire. Under-reporting remains a key limitation of self-reported alcohol intake [42], physical activity [43], smoking [44] and dietary intake [45].

## Conclusions

This study emphasizes the importance of socioeconomic factors, social support, and medical history in understanding and addressing unhealthy lifestyle behaviours among middle-aged adults. The findings suggest that middle-aged adults with lower socioeconomic status, less quality social support, and a history of disease were more likely to engage in unhealthy behaviours. Targeted interventions should consider these factors to reduce health disparities and promote healthier choices in this population.

## Supplementary Information

Additional file 1: Supplementary Material 1: Distribution of unhealthy alcohol consumption, physical inactivity, smoking and non-adherence to dietary recommendations in socioeconomic factors in 30154 participants aged 50 to 65.

Additional file 2: Supplementary Material 2: Distribution of unhealthy alcohol consumption, physical inactivity, smoking and non-adherence to dietary recommendations in social support in 30154 participants aged 50 to 65.

Additional file 3: Supplementary Material 3: Distribution of unhealthy alcohol consumption, smoking, Physical inactivity and non-adherence to dietary recommendations in self-reported history of support and family history of disease in 30154 participants aged 50 to 65.

## Data Availability

Due to the nature of the sensitive personal data and study materials, they cannot be made freely available. However, by contacting the corresponding author or study organization (www.scapis.org), procedures for sharing data, analytic methods, and study materials for reproducing the results or replicating the procedure can be arranged following Swedish legislation.

## Abbreviations

AUDIT: Alcohol Use Disorders Identification Test
CABG: Coronary Artery Bypass Graft Surgery
CI: Compatibility Intervals
COPD: Chronic Obstructive Pulmonary Disease
IRR: Incidence Rate Ratios
MD: Mean Differences
OR: Odds Ratios
PCI: Percutaneous Coronary Intervention
SD: Standard Deviation
SCAPIS: Swedish CArdioPulmonary bioImage Study
SHEI-score: Swedish Healthy Eating Index score
