# Supplementary material for: How are socioeconomic status, social support, and health history associated with unhealthy lifestyle behaviours in middle-aged adults? Results of the Swedish CArdioPulmonary bioImage Study (SCAPIS) COHORT
Source: Arch Public Health. 2025 Mar 24;83:75. doi: 10.1186/s13690-025-01513-7 (PMC11931769; doi:10.1186/s13690-025-01513-7)
Supplement: Supplementary file 1 — Additional file 1: Supplementary Material 1: Distribution of unhealthy alcohol consumption, physical inactivity, smoking and non-adherence to dietary recommendations in socioeconomic factors in 30154 participants aged 50 to 65. [file 13690_2025_1513_MOESM1_ESM.docx]

**Supplementary material 1 - Distribution of unhealthy alcohol consumption, physical inactivity, smoking and non-adherence to dietary recommendations in socioeconomic factors in 30154 participants aged 50 to 65**

|  | **Unhealthy alcohol consumption (IRR)** | | **Smoking (OR)** | | **Physical inactivity (OR)** | | **Non-adherence to dietary recommendation (MD)** | |
| --- | --- | --- | --- | --- | --- | --- | --- | --- |
| **Variable** | **Median (95 CI)^a^** | **Post. prob  >/< null^b^** | **Median (95 CI)^a^** | **Post. prob  >/< null^b^** | **Median (95 CI)^a^** | **Post. prob  >/< null^b^** | **Median (95 CI)^a^** | **Post. prob  >/< null^b^** |
| Age | 0.99 (0.99; 0.99) | > 99.9% | 0.98 (0.97; 0.99) | > 99.9% | 1.05 (1.04; 1.05) | > 99.9% | -0.02 (-0.02; -0.02) | > 99.9% |
| Man vs. Woman | 1.55 (1.53; 1.58) | > 99.9% | 1.01 (0.94; 1.08) | 58.4% | 1.01 (0.97; 1.06) | 70.7% | 0.47 (0.44; 0.5) | > 99.9% |
| Highest completed level of education: High school vs. university/college | 1.02 (1.0; 1.04) | 98.6% | 1.71 (1.57; 1.86) | > 99.9% | 1.57 (1.49; 1.66) | > 99.9% | 0.29 (0.26; 0.32) | > 99.9% |
| Highest completed level of education: Elementary vs. university/college | 1.04 (1.01; 1.08) | 98.9% | 2.58 (2.26; 2.93) | > 99.9% | 2.23 (2.02; 2.47) | > 99.9% | 0.5 (0.45; 0.56) | > 99.9% |
| Highest completed level of education: None vs. university/college | 1.03 (0.95; 1.16) | 74.8% | 3.16 (2.1; 4.64) | > 99.9% | 2.57 (1.7; 3.96) | > 99.9% | 0.55 (0.33; 0.77) | > 99.9% |
| Gainfully employed | 1.0 (0.96; 1.05) | 52.5% | 0.86 (0.73; 0.99) | 98.5% | 1.03 (0.92; 1.17) | 68.6% | -0.0 (-0.06; 0.06) | 56.4% |
| Unemployed or labor market measures | 1.06 (1.0; 1.13) | 97.7% | 1.01 (0.85; 1.2) | 55.7% | 1.12 (0.97; 1.35) | 93.5% | 0.09 (0.01; 0.19) | 98.4% |
| Old age or contractual pensioner | 1.05 (1.0; 1.11) | 96.6% | 0.97 (0.8; 1.15) | 64.2% | 0.81 (0.69; 0.95) | 99.7% | 0.02 (-0.04; 0.1) | 75.0% |
| Early retirement pension or sickness pension | 0.86 (0.81; 0.91) | > 99.9% | 1.04 (0.9; 1.23) | 72.3% | 1.22 (1.05; 1.43) | 99.6% | 0.07 (-0.0; 0.15) | 96.5% |
| Long term sick listed (more than 3 months) | 0.88 (0.82; 0.94) | > 99.9% | 1.04 (0.87; 1.28) | 67.6% | 1.24 (1.02; 1.52) | 99.0% | 0.07 (-0.02; 0.18) | 93.3% |
| Not gainfully employed – including leave of absence, parental leave, studying or training | 1.02 (0.96; 1.08) | 72.7% | 0.78 (0.58; 0.99) | 97.8% | 0.97 (0.83; 1.1) | 70.2% | -0.04 (-0.13; 0.03) | 87.2% |
| Ability to find 1800 Euro in a week for unforeseen events | 1.18 (1.13; 1.24) | > 99.9% | 0.68 (0.59; 0.79) | > 99.9% | 0.8 (0.7; 0.9) | > 99.9% | -0.18 (-0.25; -0.11) | > 99.9% |
| Difficulties managing regular expenses, last 12 months | 1.24 (1.19; 1.3) | > 99.9% | 1.41 (1.2; 1.64) | > 99.9% | 1.56 (1.37; 1.79) | > 99.9% | 0.1 (0.03; 0.17) | 99.8% |
| Own apartment vs. house | 1.04 (1.02; 1.07) | > 99.9% | 1.2 (1.09; 1.33) | > 99.9% | 1.04 (0.98; 1.1) | 90.8% | -0.04 (-0.08; -0.01) | 99.4% |
| Rental apartment vs. house | 1.03 (1.01; 1.06) | 99.2% | 1.77 (1.59; 1.97) | > 99.9% | 1.38 (1.28; 1.48) | > 99.9% | 0.04 (-0.0; 0.08) | 97.2% |
| Other vs. house | 1.11 (1.0; 1.25) | 98.0% | 1.34 (0.97; 2.06) | 95.5% | 1.27 (0.98; 1.79) | 95.8% | 0.09 (-0.03; 0.26) | 91.9% |
| Divorced vs. married | 1.04 (0.99; 1.1) | 93.7% | 1.03 (0.88; 1.27) | 65.0% | 0.95 (0.82; 1.07) | 80.2% | -0.02 (-0.09; 0.06) | 66.1% |
| Living alone vs. married | 1.0 (0.95; 1.05) | 51.3% | 1.08 (0.93; 1.34) | 83.0% | 1.13 (0.99; 1.31) | 96.4% | 0.06 (-0.01; 0.15) | 94.8% |
| Widow vs. married | 0.96 (0.88; 1.03) | 88.6% | 1.07 (0.87; 1.43) | 73.2% | 1.05 (0.9; 1.31) | 74.8% | 0.05 (-0.04; 0.17) | 83.6% |
| Not sharing a household with anybody | 1.01 (0.96; 1.05) | 60.2% | 1.01 (0.87; 1.18) | 57.7% | 1.0 (0.9; 1.11) | 51.5% | -0.0 (-0.07; 0.06) | 54.0% |
| Sharing a household withh parents, siblings, or other adults | 0.94 (0.88; 1.0) | 98.0% | 1.01 (0.84; 1.22) | 54.8% | 1.09 (0.95; 1.3) | 87.6% | 0.03 (-0.04; 0.13) | 79.5% |
| Sharing a household with a spouse or partner | 1.02 (0.98; 1.09) | 82.2% | 0.74 (0.61; 0.93) | 99.6% | 0.94 (0.81; 1.07) | 83.3% | -0.07 (-0.16; 0.01) | 96.2% |
| Sharing a household with children | 0.92 (0.9; 0.94) | > 99.9% | 0.86 (0.77; 0.95) | 99.9% | 1.02 (0.96; 1.08) | 72.0% | 0.06 (0.02; 0.1) | 99.9% |
| Born in: Europe vs. Sweden | 0.8 (0.76; 0.84) | > 99.9% | 1.17 (0.99; 1.44) | 96.4% | 1.2 (1.05; 1.39) | 99.7% | -0.04 (-0.12; 0.02) | 89.7% |
| Born in: Eastern mediterranean region vs. Sweden | 0.42 (0.35; 0.54) | > 99.9% | 1.15 (0.86; 1.89) | 82.6% | 1.26 (0.95; 2.28) | 92.6% | -0.07 (-0.29; 0.07) | 82.3% |
| Born in: Rest of world vs Sweden | 0.96 (0.84; 1.05) | 79.2% | 0.95 (0.68; 1.21) | 67.4% | 1.11 (0.92; 1.6) | 85.0% | -0.0 (-0.13; 0.12) | 52.3% |
| Mother born in: Europe vs. Sweden | 1.01 (0.97; 1.05) | 67.7% | 1.04 (0.91; 1.21) | 73.7% | 1.06 (0.97; 1.18) | 89.6% | -0.03 (-0.09; 0.02) | 87.3% |
| Mother born in: Eastern mediterranean region vs. Sweden | 0.98 (0.77; 1.13) | 64.0% | 1.13 (0.83; 2.05) | 78.3% | 1.01 (0.65; 1.37) | 52.1% | -0.04 (-0.27; 0.11) | 73.3% |
| Mother born in: Rest of world vs Sweden | 0.78 (0.67; 0.92) | 99.9% | 1.0 (0.76; 1.37) | 50.8% | 0.91 (0.62; 1.09) | 83.2% | -0.01 (-0.17; 0.11) | 59.4% |
| Father born in: Europe vs. Sweden | 0.99 (0.95; 1.03) | 71.7% | 1.15 (0.99; 1.37) | 96.2% | 1.02 (0.92; 1.14) | 67.6% | -0.07 (-0.13; -0.01) | 98.9% |
| Father born in: Eastern mediterranean region vs. Sweden | 0.96 (0.75; 1.09) | 72.3% | 1.03 (0.65; 1.54) | 57.7% | 1.01 (0.68; 1.36) | 52.2% | -0.01 (-0.19; 0.17) | 59.2% |
| Father born in: Rest of world vs Sweden | 0.89 (0.76; 1.0) | 97.2% | 0.98 (0.73; 1.25) | 59.6% | 0.99 (0.8; 1.22) | 53.1% | -0.33 (-0.5; -0.15) | > 99.9% |
| ^a^ The median of the posterior distribution over incidence rate ratios, odds ratios, and mean differences with 2.5% and 97.5% percentiles representing a compatibility interval (CI).  ^b^ The proportion of the posterior distribution less or greater than the null in the direction of the median. | | | | | | | | |
