# Supplementary material for: How are socioeconomic status, social support, and health history associated with unhealthy lifestyle behaviours in middle-aged adults? Results of the Swedish CArdioPulmonary bioImage Study (SCAPIS) COHORT
Source: Arch Public Health. 2025 Mar 24;83:75. doi: 10.1186/s13690-025-01513-7 (PMC11931769; doi:10.1186/s13690-025-01513-7)
Supplement: Supplementary file 2 — Additional file 2: Supplementary Material 2: Distribution of unhealthy alcohol consumption, physical inactivity, smoking and non-adherence to dietary recommendations in social support in 30154 participants aged 50 to 65. [file 13690_2025_1513_MOESM2_ESM.docx]

**Supplementary material 2 - Distribution of unhealthy alcohol consumption, physical inactivity, smoking and non-adherence to dietary recommendations in social support in 30154 participants aged 50 to 65**

|  | **Unhealthy alcohol consumption (IRR)** | | **Smoking (OR)** | | **Physical inactivity (OR)** | | **Non-adherence to dietary recommendations (MD)** | |
| --- | --- | --- | --- | --- | --- | --- | --- | --- |
| **Variable** | **Median (95 CI)** | **Post. prob  >/< null** | **Median (95 CI)** | **Post. prob  >/< null** | **Median (95 CI)** | **Post. prob  >/< null** | **Median (95 CI)** | **Post. prob  >/< null** |
| Age | 1.0 (0.99; 1.0) | > 99.9% | 0.99 (0.98; 0.99) | 99.9% | 1.04 (1.03; 1.05) | > 99.9% | -0.02 (-0.02; -0.02) | > 99.9% |
| Man vs. Woman | 1.55 (1.52; 1.58) | > 99.9% | 1.0 (0.93; 1.06) | 54.2% | 1.01 (0.97; 1.06) | 70.7% | 0.47 (0.44; 0.5) | > 99.9% |
| Number of acquaintances with shared interests | 1.01 (1.0; 1.02) | 97.9% | 0.9 (0.87; 0.93) | > 99.9% | 0.9 (0.88; 0.92) | > 99.9% | -0.03 (-0.05; -0.02) | > 99.9% |
| Number of people met during an ordinary week | 0.99 (0.98; 1.0) | 99.3% | 0.88 (0.85; 0.91) | > 99.9% | 0.97 (0.95; 0.99) | 99.9% | 0.0 (-0.01; 0.01) | 55.5% |
| Number of friends who can visit at any time | 1.02 (1.0; 1.03) | 99.8% | 1.11 (1.06; 1.16) | > 99.9% | 0.98 (0.95; 1.0) | 95.7% | -0.01 (-0.03; 0.0) | 94.3% |
| Number of people with whom to speak openly | 0.98 (0.97; 0.99) | 99.9% | 1.0 (0.96; 1.05) | 54.2% | 1.0 (0.98; 1.04) | 61.7% | -0.03 (-0.05; -0.01) | 99.8% |
| Number of people who can be easily asked for assistance | 1.05 (1.04; 1.07) | > 99.9% | 1.0 (0.96; 1.05) | 50.7% | 0.99 (0.96; 1.01) | 82.5% | 0.01 (-0.01; 0.03) | 83.2% |
| Number of people who can be turned to in difficulties | 0.97 (0.95; 0.98) | > 99.9% | 0.97 (0.91; 1.01) | 90.8% | 0.97 (0.94; 1.0) | 95.9% | -0.04 (-0.06; -0.02) | > 99.9% |
| No person who can provide tangible support | 1.01 (0.98; 1.05) | 75.9% | 1.05 (0.96; 1.24) | 82.3% | 1.05 (0.97; 1.19) | 87.8% | 0.01 (-0.03; 0.07) | 70.8% |
| No person who is very close | 1.02 (0.99; 1.05) | 85.9% | 1.1 (0.98; 1.28) | 93.4% | 1.05 (0.98; 1.17) | 89.6% | 0.01 (-0.03; 0.06) | 71.9% |
| No person to share feelings of happiness | 0.99 (0.96; 1.02) | 66.6% | 1.01 (0.9; 1.14) | 57.9% | 1.01 (0.95; 1.13) | 67.4% | 0.02 (-0.03; 0.09) | 79.8% |
| No person to confide in | 1.0 (0.98; 1.03) | 57.7% | 1.03 (0.95; 1.18) | 76.0% | 1.02 (0.96; 1.12) | 75.6% | 0.01 (-0.03; 0.07) | 75.5% |
| No person for comfort | 1.04 (1.01; 1.07) | 99.9% | 1.04 (0.97; 1.15) | 85.7% | 1.12 (1.04; 1.2) | 99.9% | 0.14 (0.1; 0.18) | > 99.9% |
| No people who appreciate efforts | 1.03 (1.0; 1.07) | 98.4% | 1.3 (1.14; 1.47) | > 99.9% | 1.17 (1.06; 1.29) | > 99.9% | 0.14 (0.09; 0.19) | > 99.9% |
| ^a^ The median of the posterior distribution over incidence rate ratios, odds ratios, and mean differences with 2.5% and 97.5% percentiles representing a compatibility interval (CI).  ^b^ The proportion of the posterior distribution less or greater than the null in the direction of the median. | | | | | | | | |
