# Supplementary material for: How are socioeconomic status, social support, and health history associated with unhealthy lifestyle behaviours in middle-aged adults? Results of the Swedish CArdioPulmonary bioImage Study (SCAPIS) COHORT
Source: Arch Public Health. 2025 Mar 24;83:75. doi: 10.1186/s13690-025-01513-7 (PMC11931769; doi:10.1186/s13690-025-01513-7)
Supplement: Supplementary file 3 — Additional file 3: Supplementary Material 3: Distribution of unhealthy alcohol consumption, smoking, Physical inactivity and non-adherence to dietary recommendations in self-reported history of support and family history of disease in 30154 participants aged 50 to 65. [file 13690_2025_1513_MOESM3_ESM.docx]

**Supplementary material 3 - Distribution of unhealthy alcohol consumption, smoking, Physical inactivity and non-adherence to dietary recommendations in self-reported history of disease and family history of disease in 30154 participants aged 50 to 65**

|  | **Unhealthy alcohol consumption** | | **Smoking** | | **Physical inactivity** | | **Non-adherence to dietary recommendations** | |
| --- | --- | --- | --- | --- | --- | --- | --- | --- |
| **Variable** | **Median (95 CI)** | **Post. prob  >/< null** | **Median (95 CI)** | **Post. prob  >/< null** | **Median (95 CI)** | **Post. prob  >/< null** | **Median (95 CI)** | **Post. prob  >/< null** |
| Age | 0.99 (0.99; 1.0) | > 99.9% | 0.98 (0.97; 0.99) | > 99.9% | 1.03 (1.03; 1.04) | > 99.9% | -0.02 (-0.02; -0.02) | > 99.9% |
| Man vs. Woman | 1.55 (1.52; 1.58) | > 99.9% | 0.99 (0.93; 1.05) | 60.9% | 1.03 (0.98; 1.08) | 89.2% | 0.51 (0.48; 0.54) | > 99.9% |
| Myocardial infarction, angina pectoris, atrial fibrillation, heart failure, or heart valve disease | 0.94 (0.9; 0.99) | 99.5% | 0.99 (0.85; 1.1) | 60.5% | 0.99 (0.89; 1.07) | 63.3% | -0.02 (-0.09; 0.02) | 81.9% |
| CABG, PCI intervention, peripheral artery disease intervention, or aortic intervention | 0.98 (0.91; 1.04) | 74.1% | 1.02 (0.89; 1.3) | 65.6% | 1.0 (0.88; 1.16) | 53.4% | 0.01 (-0.06; 0.1) | 59.6% |
| Stroke | 0.9 (0.82; 0.98) | 99.2% | 1.01 (0.88; 1.24) | 58.7% | 0.99 (0.86; 1.14) | 55.0% | 0.02 (-0.04; 0.13) | 72.8% |
| Hypertension | 1.08 (1.05; 1.1) | > 99.9% | 1.0 (0.93; 1.08) | 52.1% | 1.31 (1.23; 1.4) | > 99.9% | 0.09 (0.05; 0.13) | > 99.9% |
| Hyperlipidemia | 1.09 (1.05; 1.12) | > 99.9% | 1.14 (1.01; 1.3) | 98.3% | 1.02 (0.96; 1.1) | 70.9% | 0.01 (-0.02; 0.06) | 74.5% |
| COPD, chronic bronchitis, emphysema, tuberculosis, or other lung diseases | 1.14 (1.07; 1.21) | > 99.9% | 1.99 (1.63; 2.4) | > 99.9% | 1.49 (1.26; 1.78) | > 99.9% | 0.19 (0.09; 0.29) | > 99.9% |
| Asthma | 0.99 (0.96; 1.02) | 70.3% | 0.82 (0.7; 0.97) | 99.3% | 1.05 (0.98; 1.16) | 91.0% | 0.02 (-0.02; 0.07) | 79.0% |
| Sleep apnea | 1.06 (1.01; 1.1) | 99.3% | 1.02 (0.92; 1.18) | 66.6% | 1.36 (1.19; 1.55) | > 99.9% | 0.06 (-0.0; 0.14) | 96.2% |
| Celiac disease | 0.97 (0.86; 1.04) | 80.5% | 0.69 (0.28; 1.04) | 90.9% | 0.97 (0.75; 1.12) | 66.5% | -0.13 (-0.36; 0.02) | 93.8% |
| Crohn’s disease or ulcerative colitis | 1.02 (0.96; 1.1) | 70.2% | 0.98 (0.73; 1.14) | 64.6% | 1.03 (0.91; 1.26) | 71.0% | 0.03 (-0.04; 0.17) | 80.1% |
| Diabetes | 0.83 (0.79; 0.87) | > 99.9% | 1.29 (1.04; 1.57) | 99.5% | 1.43 (1.24; 1.64) | > 99.9% | -0.08 (-0.16; -0.0) | 98.1% |
| Rheumatic disease | 0.94 (0.89; 0.99) | 99.3% | 1.09 (0.97; 1.36) | 89.4% | 1.05 (0.96; 1.2) | 84.8% | 0.02 (-0.03; 0.09) | 76.9% |
| Cancer | 0.97 (0.94; 1.01) | 92.1% | 0.92 (0.77; 1.03) | 90.2% | 0.98 (0.89; 1.06) | 72.2% | -0.01 (-0.07; 0.03) | 73.0% |
| Family history of diabetes, any first degree relative | 0.94 (0.92; 0.97) | > 99.9% | 1.0 (0.93; 1.08) | 52.0% | 1.15 (1.08; 1.22) | > 99.9% | 0.02 (-0.01; 0.05) | 87.5% |
| Family history of asthma, any first degree relative | 0.99 (0.96; 1.01) | 89.7% | 0.96 (0.87; 1.03) | 86.4% | 1.1 (1.03; 1.17) | 99.9% | 0.01 (-0.02; 0.04) | 75.3% |
| Family history of bronchitis, COPD or emphysema, any first degree relative | 1.06 (1.03; 1.09) | > 99.9% | 1.18 (1.06; 1.31) | > 99.9% | 1.05 (0.98; 1.12) | 92.6% | 0.08 (0.04; 0.12) | > 99.9% |
| Family history of myocardial infarction, subject’s parent or sibling | 0.97 (0.95; 0.99) | 99.6% | 0.98 (0.91; 1.05) | 71.7% | 1.06 (1.0; 1.12) | 98.1% | -0.0 (-0.03; 0.02) | 62.1% |
| Family history of stroke, subject’s parent or sibling | 1.02 (1.0; 1.04) | 98.3% | 1.03 (0.97; 1.11) | 79.7% | 0.98 (0.93; 1.02) | 83.2% | -0.02 (-0.05; 0.01) | 87.8% |
| Family history of lung cancer, subject’s parent or sibling | 1.02 (0.99; 1.06) | 92.8% | 1.02 (0.93; 1.14) | 65.6% | 1.12 (1.02; 1.24) | 99.1% | 0.0 (-0.04; 0.05) | 59.4% |
| ^a^ The median of the posterior distribution over incidence rate ratios, odds ratios, and mean differences with 2.5% and 97.5% percentiles representing a compatibility interval (CI).  ^b^ The proportion of the posterior distribution less or greater than the null in the direction of the median. | | | | | | | | |
